# Reviews

**Published:** 1883-12-20

**Authors:** 


### *REVIEWS.*

A Practical Treatise on Materia Medica and Therapeutics, by Roberts Bartholow, M. A., M. D., LL.D., Prof. of Materia Medica and general Therapeutics, in the Jefferson Medical College, Philadelphia, etc. Fifth edition, revised and enlarged. New York: D. Appleton & Co. 1884.

Prof. Bartholow is too widely and favorably known to the Profession to require any special remarks upon his ability as a thinker and writer. This new edition he tells us is made necessary by the late revision of the United States Pharmacopiœa, and the author has adapted his work to the official standard, incorporating all the late improvements in the science and art of Therapeutics. Many valuable additions have also been made and parts rewritten; practical utility being kept in view.

Seventy pages have been added, and by sacrifice of references, etc., much additional matter has been given without loss of useful material and without increasing space. The work, as now presented, is neatly and well gotten out, and contains 738 octavo pages.

The Clinical Index at close of volume will be found very convenient and useful, and the general index is very satisfactory.

The style of the writer is forcible, and compact; nothing irrelevant, and yet sufficiently full for all pvrposes. An admirable text book, and being complete and well up with the late advances, is also an excellent reference book for the practitioner.

A Digest of Materia Medica and Pharmacy, forming a complete Pharmacopiœa for the use of physicians, Druggists and Students, by Albert Merrell, M.D., Prof. of Chemistry, Pharmacy, and Toxicology, in the American Medical College, St. Louis, Mo, member of the State Board of Health of Missouri. Philadelphia: P. Blakiston, Son & Co, 1012 Walnut street, 1883.

The work contains 512 octavo pages and is gotten out in beautiful style and excellent taste, the style of the writer being easy and graceful, yet terse, pointed and practical. The writer, we suppose, is an "Eclectic," as he is connected with a school of that order, but he makes a good and liberal impression in his preface, the tone and sentiments of which are not objectionable, as he well says that "The spirit of the modern investigator, in every field of science, is selective, eclective or truly eclective, evincing no respect for theory or practice, however aged, which does not invite the most rigid examination before claiming acceptance." We of the regular school find no fault with this sentiment and claim that we are truly eclectic in the proper sense of that word, and will not be slow to avail ourselves of anything good from any and any source.

We find in this book many of the new remedies intelligently described, from a practical stand-point, and the dose and therapeutical action well and truthfully presented, and we are willing to accord to the author the distinction, so far as we can judge, of presenting in this book the best work which we have yet seen from the school or system of medicine which he represents.

